# In Vitro Co-Delivery Evaluation of Novel Pegylated Nano-Liposomal Herbal Drugs of Silibinin and Glycyrrhizic Acid (Nano-Phytosome) to Hepatocellular Carcinoma Cells 

**DOI:** 10.22074/cellj.2016.4308

**Published:** 2016-05-30

**Authors:** Mohammad Mahdi Ochi, Ghasem Amoabediny, Seyed Mahdi Rezayat, Azim Akbarzadeh, Bahman Ebrahimi

**Affiliations:** 1 Department of Life Science Engineering, Faculty of New Sciences and Technologies, University of Tehran, Tehran, Iran; 2 Department of Nano Biotechnology, Research Center for New Technologies in Life Science Engineering, University of Tehran, Tehran, Iran; 3 Department of Biotechnology and Pharmaceutical Engineering, School of Engineering, University of Tehran, Tehran, Iran; 4 School of Advanced Technologies in Medicine, Tehran University of Medical Sciences, Tehran, Iran; 5 Pasteur Institute of Iran (IPI), Tehran, Iran

**Keywords:** Encapsulation, Silibinin, Glycyrrhizic Acid, Hepatocellular Carcinoma

## Abstract

**Objective:**

This study aimed to evaluate a co-encapsulated pegylated nano-liposome system based on two herbal anti-tumor drugs, silibinin and glycyrrhizic acid, for delivery to a hepatocellular carcinoma (HCC) cell line (HepG2).

**Materials and Methods:**

In this experimental study, co-encapsulated nano-liposomes by the thin layer film hydration method with HEPES buffer and sonication at 60% amplitude. Liposomes that co-encapsulated silibinin and glycyrrhizic acid were prepared with a specified molar ratio of dipalmitoylphosphatidylcholine (DPPC), cholesterol (CHOL), and methoxy-polyethylene glycol 2000 (PEG2000)–derived distearoyl phosphatidylethanolamine (mPEG2000-DSPE). We used the MTT technique to assess cytotoxicity for various concentrations of co-encapsulated nano-liposomes, free silibinin (25% w/v) and glycyrrhizic acid (75% w/v) on HepG2 and fibroblast cell lines over a 48-hour period.

**Results:**

Formulation of pegylated nano-liposomes showed a narrow size distribution with an average diameter of 46.3 nm. The encapsulation efficiency (EE) for silibinin was 24.37%, whereas for glycyrrhizic acid it was 68.78%. Results of *in vitro* cytotoxicity showed significantly greater co-encapsulated nano-liposomes on the HepG2 cell line compared to the fibroblast cell line. The half maximal inhibitory concentration (IC_50_) for co-encapsulated pegylated nanoliposomal herbal drugs was 48.68 µg/ml and free silibinin with glycyrrhizic acid was 485.45 µg/ml on the HepG2 cell line.

**Conclusion:**

This *in vitro* study showed that nano-liposome encapsulation of silibinin with glycyrrhizic acid increased the biological activity of free drugs, increased the stability of silibinin, and synergized the therapeutic effect of silibinin with glycyrrhizic acid. The IC_50_ of the co-encapsulated nano-liposomes was lower than the combination of free silibinin and glycyrrhizic acid on the HepG2 cell line.

## Introduction

Worldwide, cancer is recognized as a deadly disease; in this regard, hepatocellular carcinoma (HCC) is the sixth most common cancer in the world. However because of poor prognosis, HCC is considered the third cause of cancer related deaths (1). Conventional anticancer drugs cause various side effects that result from non-selective toxicity and distribution of the drug to normal cells (2).

Nano-liposomes are drug carriers with lipoid membranes used to improve drug delivery. Nanophytosomes are nano-phyto-liposome vesicles formed due the interaction of hydrogen bonds between phospholipids of the lipid membrane and phytomolecules for improving the delivery of therapeutic agents. Specifically, the anti-cancer potency of drugs increases with decreasing size of the liposome (3).

Most studies of phyto-liposomes focus on the liver-protectant flavonoid (silymarin mixture) from the milk thistle [Silybum marianum (L.)], a medicinal plant from Iran (4). Silymarin properties include immunomodulation, anti- fibrotic, membrane stabilizing, anti-oxidative, anti-inflammatory, and liver regeneration that play essential roles in experimental models of liver diseases (5). The components of silymarin are used for hepatoprotection, while having poor aqueous solubility and low bioavailability. *In vivo* studies regarding oral consumption of powdered components of silymarin have shown ng/ml elevated concentrations in the blood plasma (6, 7). Various clinical and pharmacological effects of silymarin have been reported, such as targeting cancer cell metastasis. Unfortunately, silymarin’s poor solubility in water and oil results in permeation through the intestinal epithelial membrane (8). The dried extracts from milk thistle seeds contain approximately 60% silymarin. Silymarin consists of silibinin (~50 to 60%), isosilibinin (~5%), silichristin (~20%), and silidanin (~10%). Silibinin is the most important antioxidant substance and the main biological active compound of silymarin (9). Silibinin is an anti-cancer drug; its antitumor efficacy lies mainly in decreasing N-nitrosodiethylamine in liver cancer cells (10). An advantage of the interaction between hydroxyl groups and the silibinin molecule is the rational identification of suitable sites for silibinin derivation while maintaining the biological activity of the resultant conjugates (11). Chu et al. (12) have shown that silibinin exerted a prohibitive effect on the encroachment and intrusion of advanced metastatic A549 cells, but had little effect on adhesion. Silymarin mediated these effects by decreasing the expressions of matrix metalloproteases (MMP)-2 and u-PA, and enhancing the expression of tissue inhibitor of MMP (TIMP)-2 (12). Oral absorption of silibinin is limited and solubility of silibinin is poor in water and lipid media (13).

Therefore, there is a strong need to develop new drug delivery systems that improve the solubility and bioavailability of silibinin. In previous studies, silymarin has been encapsulated to liposomes with a mixture of lecithin, cholesterol (CHOL), stearyl amine, and Tween 20 at a 9:1:1:0.5 molar ratio as the lipophilic phase with a particle size of 800 nm (14).

In addition, we studied glycyrrhizic acid, an herbal drug obtained from *Glycyrrhiza glabra (L.)*. Glycyrrhizic acid possesses hepatoprotector, anti-viral, anti-tumor, and anti-inflammatory properties. The anti-tumor efficacy of glycyrrhizic acid is due to inhibition of MMPs and protection of DNA in cancer cells. In previous studies such as Japan’s drug of neo-minophagen c based glycyrrhizic acid was used for hepatic disease, Italy’s drug of glycyrrhetinic acid phytosome was prepared based on liposomal glycyrrhizic acid, and Russia’s drug of phosphoglive includes vegetative phospholipids with glycyrrhizic acid (15).

All previous studies and products focused on preparation of liposome based on one herbal drug, either glycyrrhizic acid or silymarin but in this study, we co-entrapped two herbal drugs (silibinin and glycyrrhizic acid) to a pegylated nano-liposome in order to assess the *in vitro* cytotoxicity of co-encapsulated nano-liposomes on a liver cancer cell line (HepG2).

## Materials and Methods

### Materials

Dipalmitoylphosphatidylcholine (DPPC) and methoxy-polyethylene glycol (PEG2000)-derivatized distearoyl phosphatidylethanolamine (mPEG2000-DSPE) were obtained from Lipoid GmbH (Ludwigshafen, Germany). CHOL, silibinin and HEPES buffer (10 mM, pH=5.5) were purchased from Sigma-Aldrich (St. Louis, MO, USA). Glycyrrhizic acid was purchased from Shirin Darou co. (Shiraz, Iran). Deionized water was used throughout the experiments. The *in vitro* release measurement was carried out at pH=7.4 in phosphate buffer and at pH=5.5 in HEPES buffer at 37˚C. Maltose, etha- nol and the remainder of chemicals used in this study were analytical grade quality. HepG2 and fibroblast cell lines were supplied by the Pasteur Institute of Iran.

### Preparation of co-encapsulated nano-liposomes

**Fig.1:**
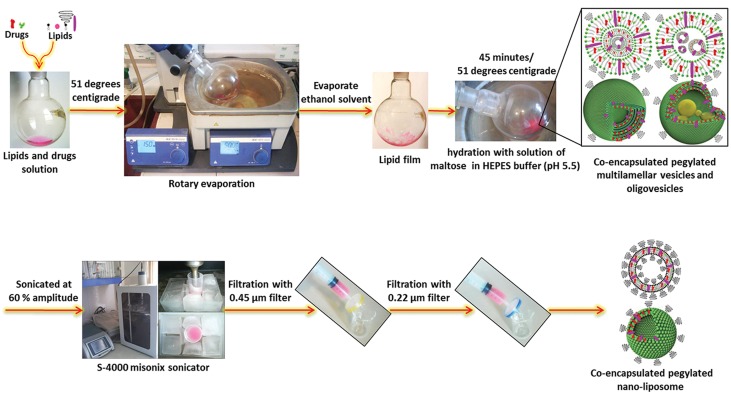
Schematic model for the preparation process of co-encapsulated nano-liposomes.

We prepared the co-encapsulated nano-liposomes by the thin layer film hydration method with HEPES buffer and sonication. First, we dissolved a mixture of DPPC, CHOL, and mPEG2000-DSPE at a specified molar ratio with two herbal drugs of silibinin and glycyrrhizic acid at a 1.74:1 molar ratio, as the lipophilic phase in absolute ethanol. The organic solvent was evaporated using a rotary evaporator to produce a thin lipid film. Before hydration, the lipid film was flushed with nitrogen. Liposomes were formed by hydration of the lipid film with a solution that contained maltose in HEPES buffer (10 mM, pH=5.5) as the hydrophilic phase and heated to 51˚C. The mean liposome diameter decreased with sonication. Multilamellar vesicles were sonicated at 60% amplitude for 10 minutes by an S-4000 Misonix sonicator and filtered for synthesis of small unilamellar vesicles (SUV) or nano-liposomes (Fig.1). The suspension was then freeze-dried using an Alpha1-2LD plus Christ Freez Dryer at −48˚C for 48 hours.

### Morphology of multilamellar vesicles

The morphology (structure and size) of multilamellar vesicles was analyzed by light microscopy.

### Size and zeta potential characterization of co-encapsulated nano-liposomes 

The particle size and zeta potential of the coencapsulated nano-liposomes were determined by dynamic light scattering (DLS) and a zeta analyzer using a Brookhaven BI-90 particle size and zeta analyzer V7.2 (Brookhaven Instruments, Holtsville, USA). 

### Measurement of encapsulation efficiency

We used HPLC to determine the encapsulation efficiency of the nano-liposomes that coencapsulated silibinin and glycyrrhizic acid. A reverse phase of the C18 column was used. The mobile phase consisted of a mixture of acetonitrile and phosphoric acid (0.1%), (51:49 v/v) delivered at a flow rate of 1.00 ml/minute with a pump (WellChrom K-1000, Knauer, Germany) (16, 17). First, to remove all free drugs, the coencapsulated nano-liposomes were dialyzed with a Spectra/Pore® Dialysis membrane (12000-14000 Da molecular weight cutoff) against the buffer. We added 200 μl of methanol to 100 μl aliquots of the extruded suspension in tubes and mixed the solution, followed by sonication for 10 minutes. A total of 60 μl of the supernatant was analyzed by the high performance liquid chromatography (HPLC) system. The absorbance of silibinin and glycyrrhizic acid were measured in a UV/V is spectrometer by HPLC in the range of 200-350 nm. Column elute was monitored spectrophotometrically at a 240 nm wavelength with a UV detector (WellChrom K-2500, Knauer, Germany). The calibration curve of silibinin and glycyrrhizic acid were linear over the range of standard concentrations 0.125, 0.25, and 0.5 mg/ ml with a correlation coefficient of R2>0.97 and R2>0.99, respectively.

In order to determine encapsulation efficiency (EE), we used the following equation (1):

$$\mathrm{Encapsulation efficiency (\%)}=\frac{\mathrm{Drug in liposome (mg)}}{\mathrm{Total of drug (mg)}}\times 100$$

### In vitro silibinin and glycyrrhizic acid release

Drug release rate was measured by HPLC. The ability to release silibinin and glycyrrhizic acid at pH=7.4 was evaluated. We placed 1 mL of liposomal suspension into a dialysis bag of cellulose that had a 12000-14000 Da molecular weight cutoff (Membrane Filtration Products, Inc.) The dialysis bags were placed in 50 mL of phosphate-buffered saline (PBS), pH=7.4, and the media were stirred with a magnetic bar at 100 rpm at 37˚C. At different time points, we removed 200 μL of the suspension for HPLC analysis in the range of 240 nm.

### Stability study

The stability of the liposomal suspension was evaluated after 3 months of storage at 4˚C. The particle size distribution, morphology and drug EE of the samples were determined as a function of the storage time.

### Morphology of co-encapsulated pegylated nano-liposomes 

The morphology (shape and size) of the nanoliposomes was examined by scanning electron microscope (SEM, KYKY-EM3200-30KV, China) at an acceleration voltage of 25 kV. The size and formation of nano-liposomes membranes with negative-stain was verified through their morphological aspect, as determined by transmission electron microscope (TEM, ZEISS, EM 10, Germany). Samples were then observed in a microscope at the accelerating voltage of 150 kV.

### ATR-Fourier transform infrared spectroscopy study of co-encapsulated nano-liposomes

The co-encapsulated pegylated nano-liposomes were analyzed by ATR-Fourier transform infrared spectroscopy (ATR-FTIR) and FTIR (Tensor 27 FTIR spectrophotometer, Bruker Corp., Germany) to determine the pegylating surface of nanoliposomes.

### Cytotoxicity assay

We decanted 100 µl of suspension that contained 10000 cells from the cultured HepG_2_ and fibroblast cells into 96-well plates, which were incubated (5% CO_2_ and 37˚C) separately. The superT - natant was removed after 24 hours, after which we poured different concentrations of pegylated nanoliposomal silibinin (25% w/v) and glycyrrhizic acid (75% w/v), free silibinin (25% w/v) with glycyrrhizic acid (75% w/v), and their controls on the cells, which were then incubated for 48 hours. Subsequently, the supernatant was removed and 100 µl of MTT solution (0.5 mg/ml) was added. After a 3-hour incubation period, we observed a purple color which indicated the formation of formazan. The mixture was then dissolved in viable cells in 100 µl of isopropanol. Light absorbance was measured at 540 nm by a Power wave XS spectrophotometer (BioTek Instruments, USA) and the IC_50_ was calculated using the Pharmacologic Calculation System (Pharm PCS) statistical package (Springer Verlag, USA).

### Statistical analysis

One-way analysis of variance (ANOVA) was performed on the data to assess the impact of the formulation variables on the results. P<0.05 were considered significant. All calculations were performed using the statistical software program SAS 9.1 (SAS Institute, Cary, NC, USA).

## Results

### Morphologies of silibinin and glycyrrhizic acid multilamellar vesicles

**Fig.2:**
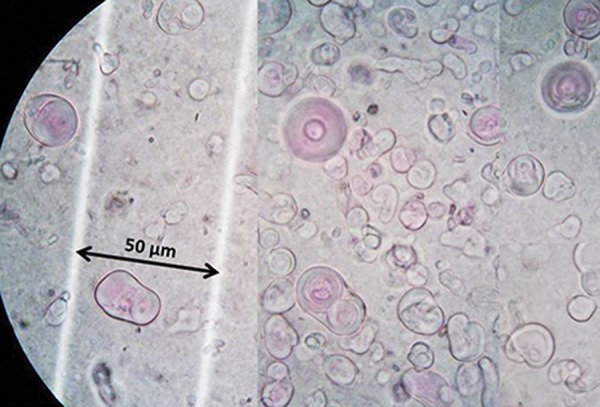
Light microscopic images of co-encapsulated multilamellar vesicles from the same samples before decreasing the size of the liposomes.

We analyzed the silibinin and glycyrrhizic acid multilamellar vesicles images via light microscopy. There were multi membranes of liposomal herbal drugs that had micrometric sizes before sonication and synthesis of SUV (Fig.2).

### Characterization of co-encapsulated nanoliposome size and zeta potential

**Fig.3:**
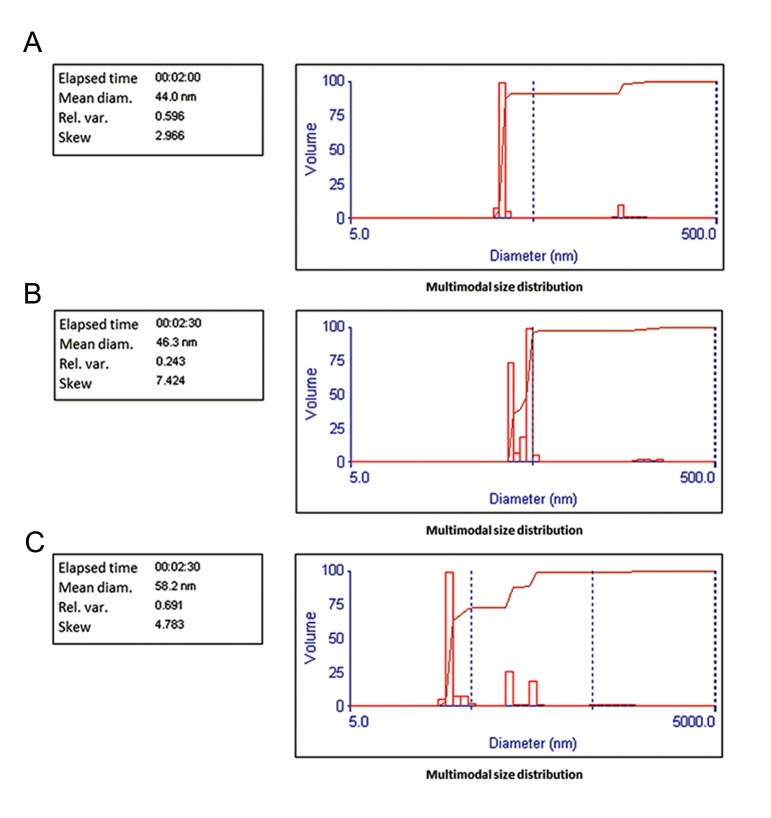
Size of: A. Nano-liposome without herbal drugs, B. Co-encapsulated nano-liposomes after 3 days, and C. Co-encapsulated nanoliposomes after 3 months.

**Fig.4:**
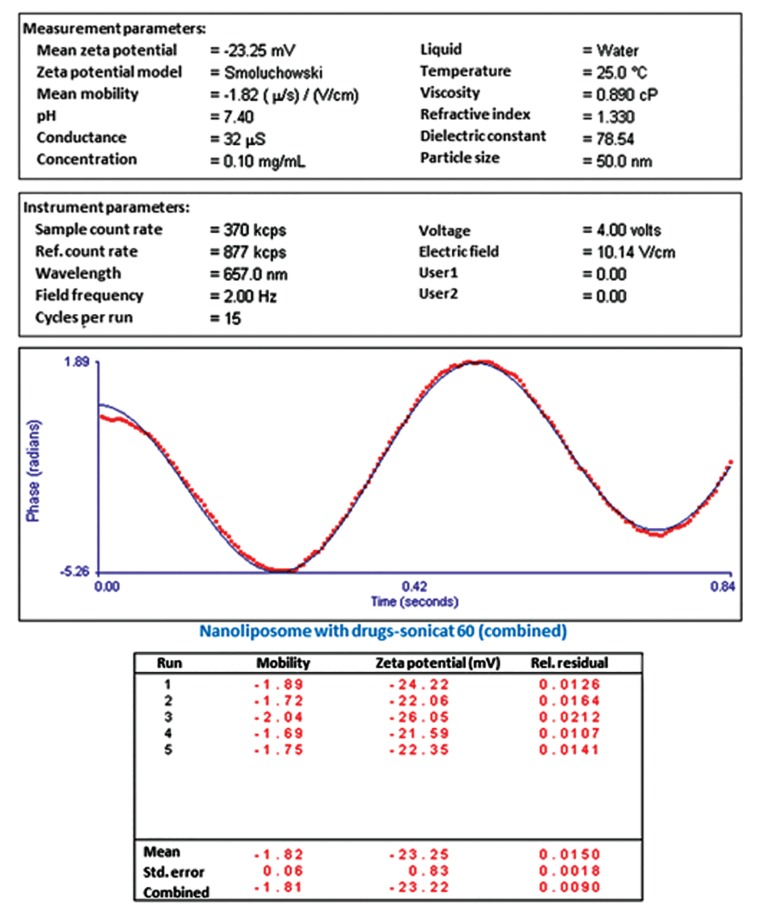
Zeta potential of co-encapsulated nano-liposomes after 3 days.

The formulation of pegylated nano-liposomes sonicated at 60% amplitude showed a narrow size distribution with an average diameter of 46.3 nm (Fig.3). The zeta potential of the co-encapsulated nano-phytosome was -23.25 mV (Fig.4).

We observed that the zeta potential of the co-encapsulated nano-phytosome had sufficient charge to inhibit aggregation of the liposomes. Loading of silibinin and glycyrrhizic acid increased the mean diameter and decreased zeta potential negative charge of the nano-liposome, respectively. The results of the mean diameter and zeta potential were obtained from approximately 150 individual liposomes.

### Encapsulation efficiency for silibinin and glycyrrhizic acid in pegylated nano-liposomes 

**Fig.5:**
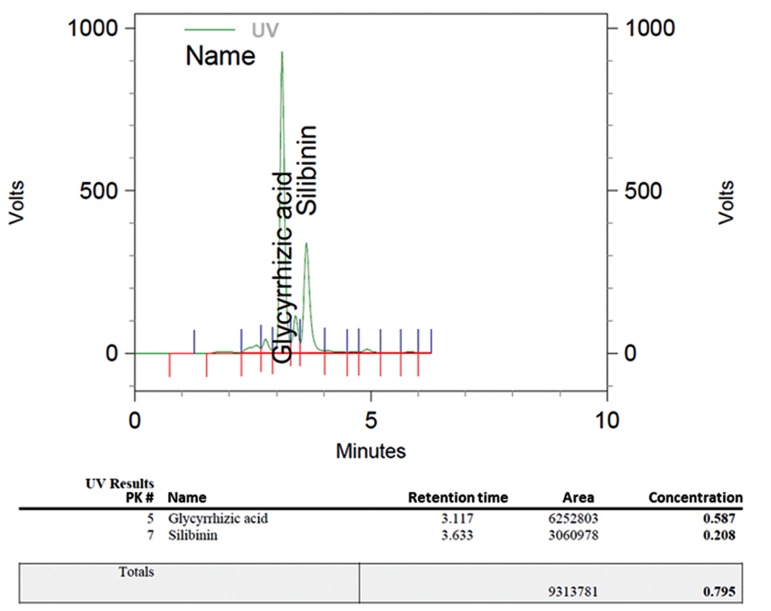
HPLC graph of silibinin and glycyrrhizic acid loaded in the nano-phytosomes. HPLC; High performance liquid chromatography.

We used HPLC to measure EE. Silibinin and glycyrrhizic acid absorbance peaks were found at a wave length of 240 nm. The EE for silibinin was approximately 24.37%, whereas for glycyrrhizic acid it was approximately 68.78%. Figure 5 shows the HPLC graph of silibinin and glycyrrhizic acid loaded in the nano-phytosomes.

### In vitro silibinin and glycyrrhizic acid release

**Fig.6:**
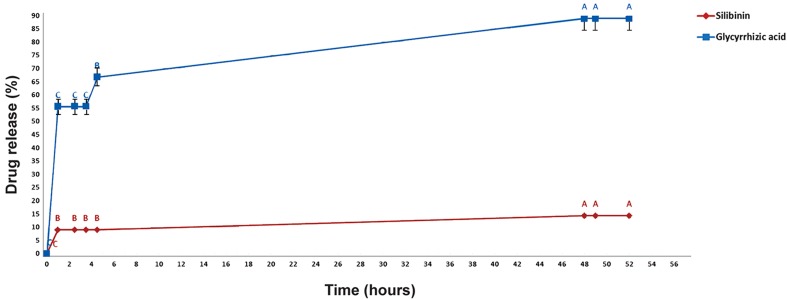
In vitro silibinin and glycyrrhizic acid release. Values with different letters demonstrate significant variance (P<0.05) from each other (n=3).

According to HPLC analyses, 14% (W/W) of silibinin and 88% (W/W) of glycyrrhizic acid were released after 48 hours. Release rate decreased over time (Fig.6). As seen in Figure 6, data with different letters show significant differences (P<0.05) from each other.

### Stability study

**Fig.7:**
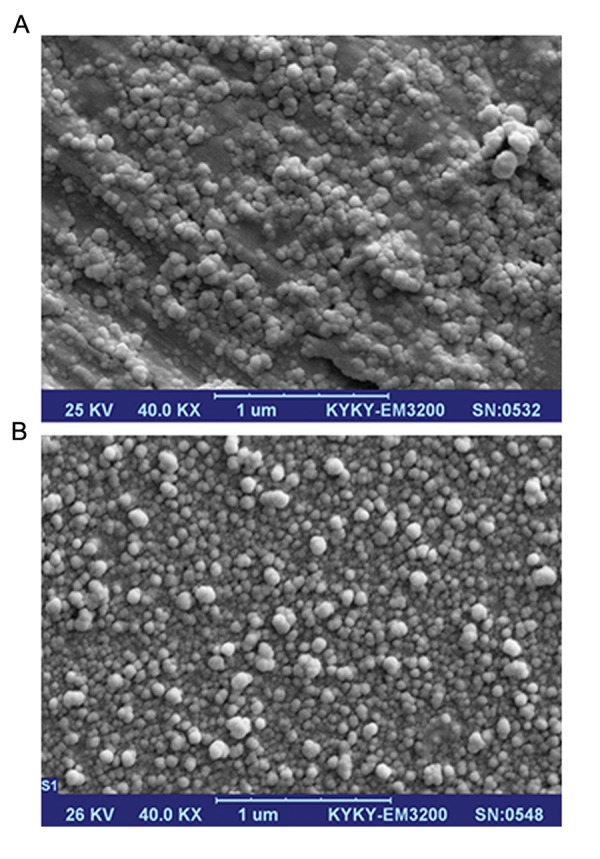
A. Scanning electron microscope (SEM) photograph of co-encapsulated nano-liposomes and B. SEM photograph of co-encapsulated nano-liposomes after 3 months.

Results of the zetasizer tests confirmed the stability of the liposomal suspension. There were negligible changes in size distribution and zeta potential of the liposomal suspension after storage at 4˚C for 3 months (Table 1). Results of SEM analysis of the liposomal suspension after storage for 3 months showed that the co-encapsulated nano-liposomes had an approximate mean diameter of 55 nm (Fig.7). The EE for liposomal silibinin and glycyrrhizic acid after storage in suspension form at 4˚C for 3 months were approximately 19.14% for silibinin and 26.75% for glycrrhizic acid.

**Table 1:** Size and zeta potential stability of liposomal suspension (about 150 individual liposomes) after storage for 3 months (mean ± SE)


Liposomal suspension	Mean zeta potential (mV)	Mean particle size (nm)

Nano-liposome without herbal drugs after 3 days	44 ± 2.2	-31.66 ± 0.39
Co-encapsulated nano-liposomes after 3 days	46.3 ± 0.4	-23.25 ± 0.83
Co-encapsulated nano-liposomes after 3 months	-25.52 ± 0.97	58.2 ± 3.8


### Morphology of co-encapsulated pegylated nano-liposomes

**Fig.8:**
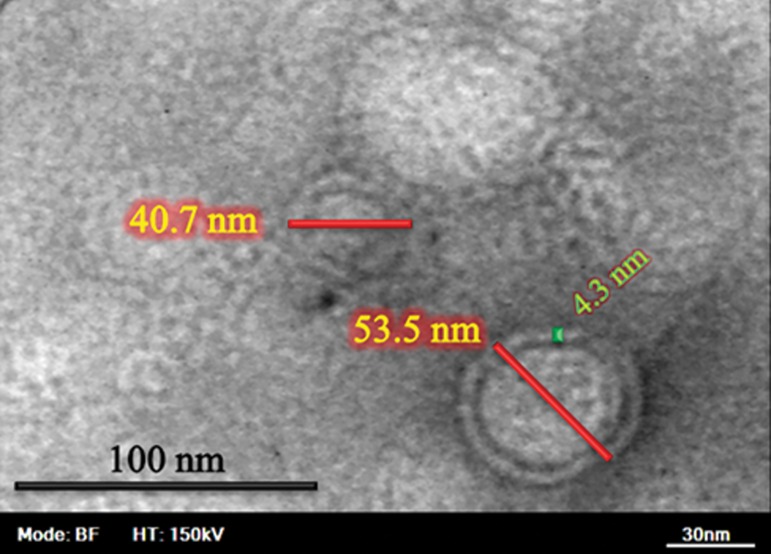
Transmission electron microscope (TEM) photograph of co-encapsulated nano-liposomes.

SEM analyses of the surface morphology of the nano-liposomes showed that the co-encapsulated nano-liposomes had a mean diameter of 43 nm (Fig.7). The morphology of the nanoliposomes was determined by TEM. Results of TEM showed that the range of diameter of coencapsulated nano-liposomes was about 40 to 60 nm (Fig.8).

### ATR-Fourier transform infrared spectroscopy study on co-encapsulated nano-liposomes

**Fig.9:**
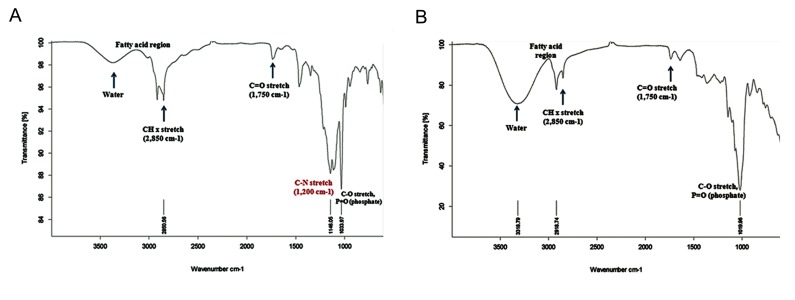
A. ATR-Fourier transform infrared spectroscopy (ATR-FTIR) of methoxy-polyethylene glycol 2000 (PEG2000)–derived distearoyl phosphatidylethanolamine mPEG-DSPE and B. ATR-FTIR of co-encapsulated nano-liposomes.

The co-encapsulated nano-liposomes were analyzed by ATR-FTIR. FTIR results demonstrated a pegylating surface of the co-encapsulated nano-liposomes. Absorption bonds of MPEG-DSPE emerged at wave- numbers 1033 cm^-1^, 1750 cm^-1^, and 2850 cm^-1^ (the distance covered by light in one second). According to Figure 9, there were negligible alterations in chemical bonds of the co-encapsulated nano-liposomes which showed that the surfaces of the co-encapsulated nano- liposomes were pegylated. 

### Cytotoxicity

**Fig.10:**
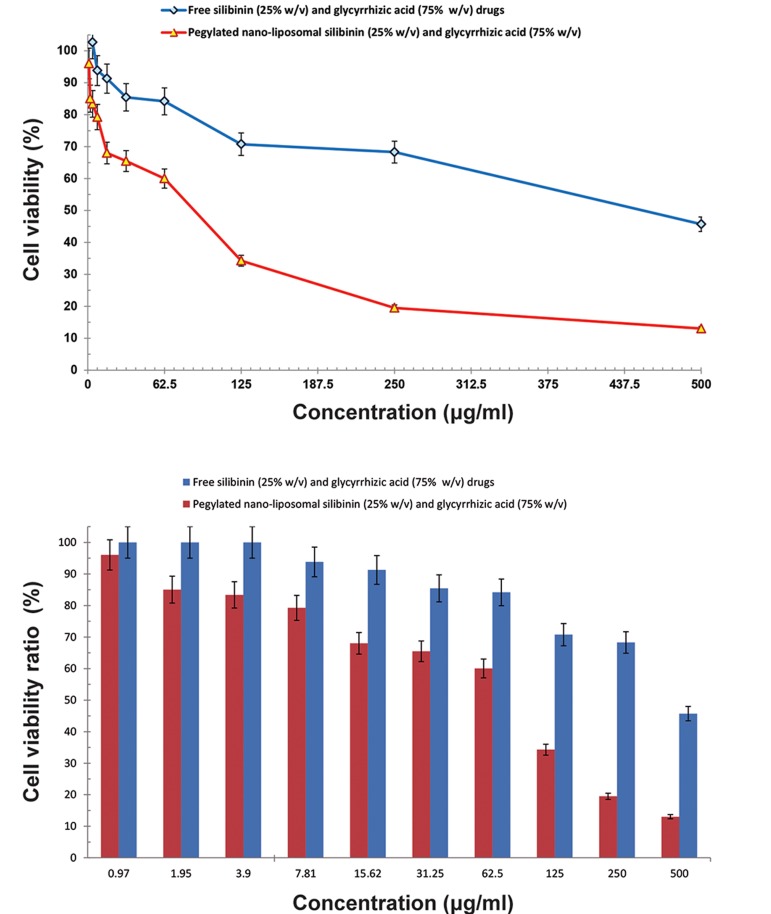
Cell viability of co-encapsulated nano-liposomes, free silibinin (25% w/v) and glycyrrhizic acid (75% w/v) on the HepG2 cell line. Values are expressed as mean ± SD. Error bars with 5% value.

**Fig.11:**
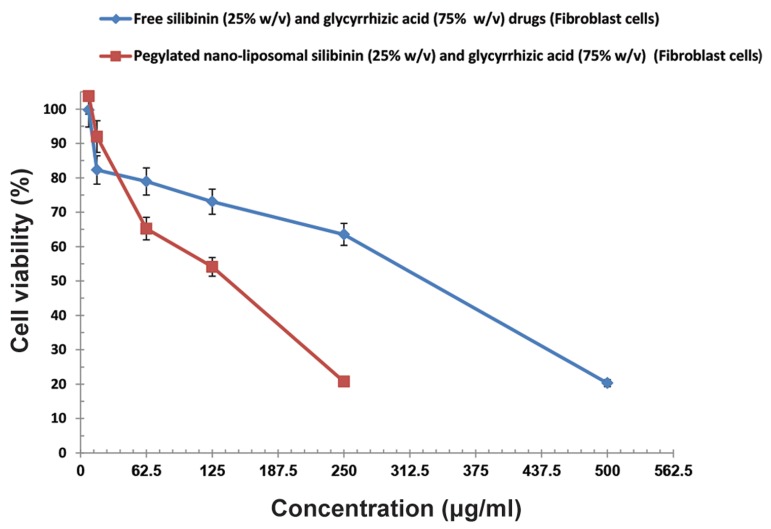
Cell viability of co-encapsulated nano-liposomes, free silibinin (25% w/v) and glycyrrhizic acid (75% w/v) on a fibroblast cell line. Values are expressed as mean ± SD.

**Fig.12:**
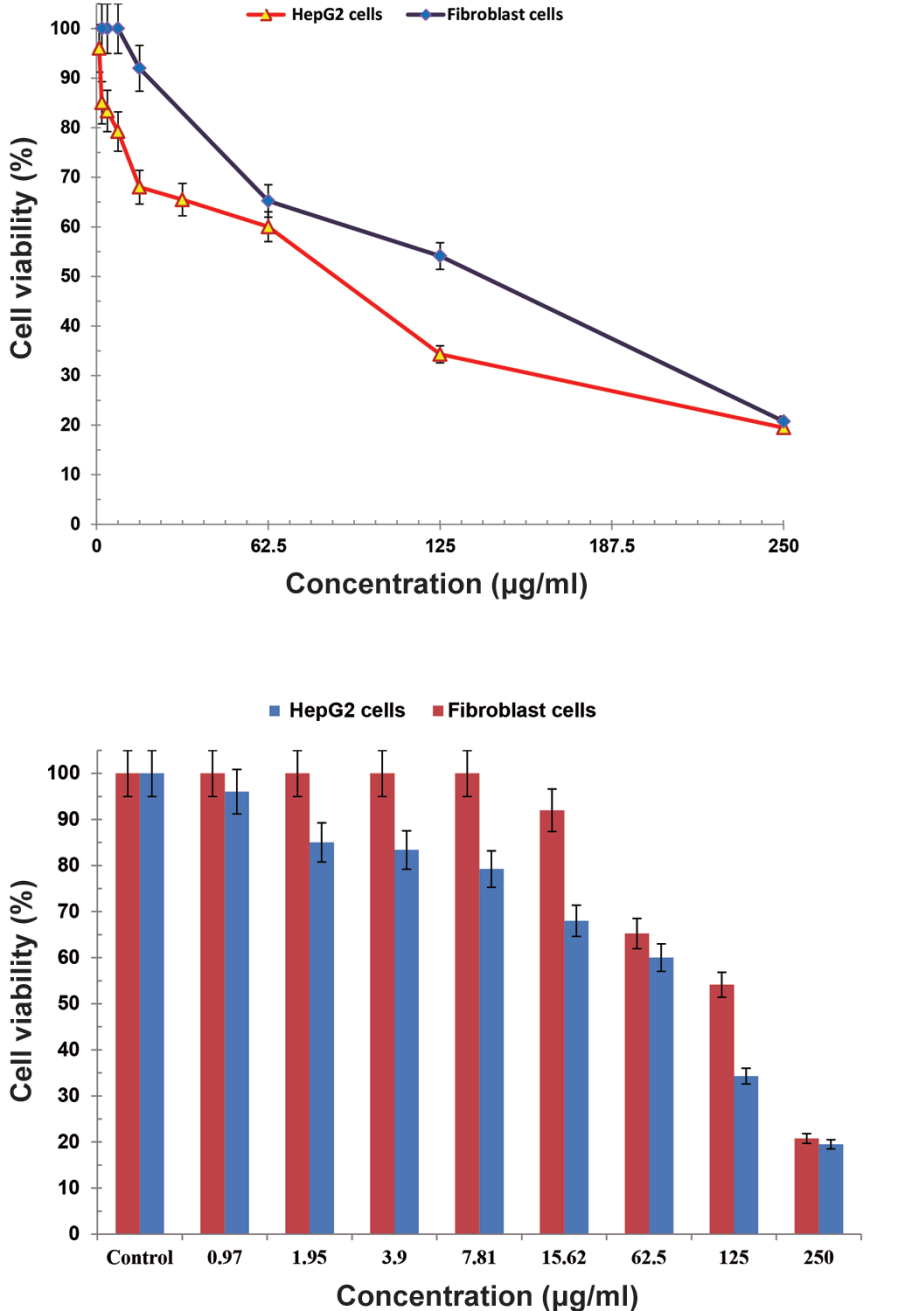
Cell viability of co-encapsulated nano-liposomes on HepG2 and fibroblast cell lines. Values are expressed as mean ± SD. Error bars with 5% value.

**Fig.13:**
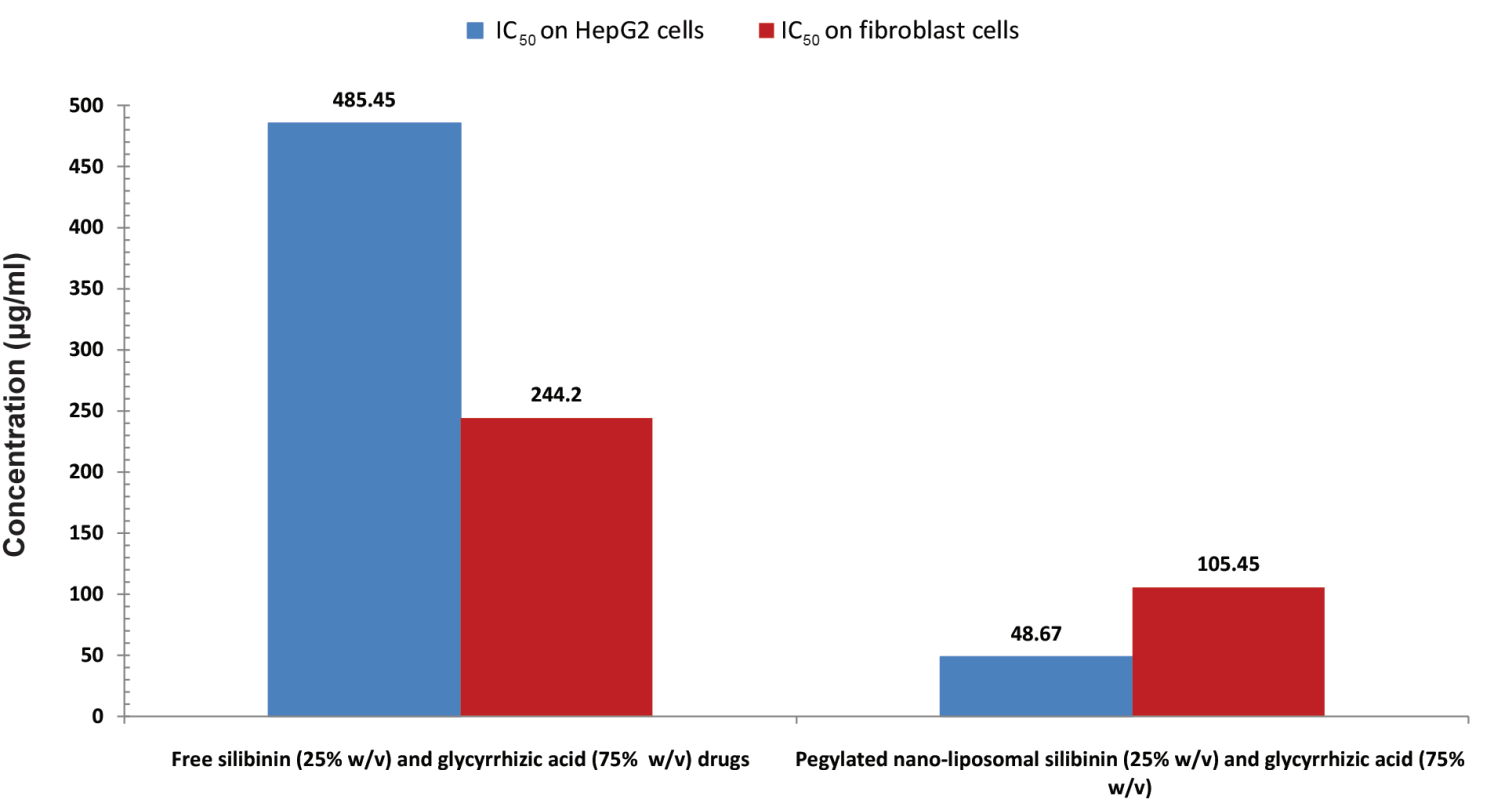
The half maximal inhibitory concentration (IC_50_) of co-encapsulated nano-liposomes, free silibinin (25% w/v) and glycyrrhizic acid (75% w/v) on HepG2 and fibroblast cells. Values are expressed as mean ± SD.

We sought to examine the presence of any drug toxicity at different concentrations based on the MTT technique. We observed the highest cytotoxicity at the time of maximum release which occurred in the first 48 hours. Cell viability of co-encapsulated nanoliposomes and free silibinin (25% w/v) and glycyrrhizic acid (75% w/v) drugs on HepG2 cell line and fibroblast cell line are shown in Figures 10 and 11, respectively. Cell viability comparison of co-encapsulated nano-liposomes on HepG2 cell line and fibroblast is shown in Figure 12. The IC_50_ for pegylated nanoliposomal silibinin and glycyrrhizic acid was 48.67 µg/ml; for free silibinin and glycyrrhizic acid, this value was 485.45 µg/ml in the HepG2 cell line. In the fibroblast cell line, the IC_50_ for pegylated nanoliposomal silibinin and glycyrrhizic acid was 105.45 µg/ml, whereas the free silibinin and glycyrrhizic acid IC_50_ was 244.2 µg/ml (Fig.13). The results showed that the cytotoxicity of pegylated nanoliposomal silibinin and glycyrrhizic acid was about ten times greater than the cytotoxicity of free herbal drugs on the HepG2 cell line. The cytotoxicity of pegylated nanoliposomal silibinin and glycyrrhizic acid on the HepG2 cell line was approximately two times greater than the cytotoxicity on the fibroblast cell line.

## Discussion

In this study, there was significantly greater in vitro cytotoxicity in the co-encapsulated nanoliposome on HepG2 cells compared to the fibroblast cell line. The cytotoxicity of co-encapsulated pegylated nano-liposome was ten times greater than free silibinin (25% w/v) and glycyrrhizic acid (75% w/v) on the liver cancer cells (HepG2 cell line).

Nano-phyto liposomes (nano-phytosome) are innovative formulations that improve bioavailability of hydrophilic flavonoids. It has been demonstrated that polar phyto constituents such as flavonoids when mixed with phospholipids (e.g., phosphatidylcholine) generate a new drug delivery system called nano-phyto liposome with a better absorption profile and more suitable lipid solubility which make them competent to cross biological membranes (4). Bombardelli et al. (18) have reported that phyto liposomes increased the specific activity of silymarin and had greater durability and stability than the free constituents. With respect to the antioxidant percentage and free radical clean- ing properties, in humans liposomal silibinin effectively is absorbed into the target tissue of liver. The results have suggested that the absorbency of liposomal silibinin is approximately 7 times greater than the absorbency of free silibinin (6). The anti-hepatotoxic activity of liposomal silymarin is better than silymarin alone and can protect broiler chicks against the toxic effects of aflatoxin B1 (19). Yanyu et al. (20) have prepared a silibinin-phospholipid complex. Due to an impressive improvement of the lipophilic, oral administration of phyto liposomal silibinin increased its bioavailability in rats and had an improved biological effect. Different clinical studies have shown the bioavailability and pharmacological safety of silymarin. Experiments are now in progress to demonstrate the clinical influence of silymarin on different cancers (21). Silibinin is the most important anti-tumor substance of the silymarin compound and largely responsible for its antihepatotoxic activity (8-10). Components of silymarin (such as silibinin) have poor solubility in aqueous and lipid and low bioavailability (6, 7, 13).

Therefore, there is a strong need to develop new drug delivery systems such as phyto-liposomes to improve both the solubility and synergize the therapeutic effect and bioavailability of silibinin. In this study, we have co-encapsulated two herbal anti-tumor drugs, silibinin and glycyrrhizic acid, to a pegylated nano-liposome. The EE for silibinin was greater than 24% and glycyrrhizic acid was over 68%. In this study, the two herbal drugs were co-entrapped to nano-phyto liposome by hydrogen bond interactions between the phospholipids and the herbal drugs. These nano-phyto liposomes could improve the delivery of phytomolecules.

El-Samaligy et al. (14) prepared liposome based on one herbal drug, silymarin, that had a mean particle size of 800 nm. Archakov et al. prepared phosphoglive using vegetative phospholipids with glycyrrhizic acid and reported a mean particle size less than 100 nm (15). All previous studies and products dealt with preparation of liposome based on one herbal drug, either glycyrrhizic acid or silymarin. However, the present study investigated co-entrapping two herbal drugs and the formulation of pegylated nano-liposomes. We observed a narrow size distribution with diameters of approximately 40 to 50 nm.

In this study, SEM photographs showed that the mean diameter of the co-encapsulated nanoliposomes was 43 nm. We have observed that the zeta potential for the herbal drug loaded liposomes was -23.25 mV. A zeta potential greater than 30 mV is necessary for effective stability and to inhibit aggregation. In the current study, we have observed that the zeta potential of the co-encapsulated nano-phytosomes had sufficient charge to inhibit liposome aggregation.

The size stability of the co-encapsulated nanoliposomes in buffer solution (4˚C) was suitable; the mean diameter of co-encapsulated nano-liposomes after 3 months was 58.2 nm. A stability study showed that the EE of liposomal suspension changed dramatically after 3 months. For increased stability of herbal drug entrapment, it was suggested that the nano-liposomal suspension should be freeze-dried after which the co-encapsulated nano-liposome stability would be analyzed after lyophilizing.

In this study, ATR-FTIR results of co-encapsulated nano-liposomes demonstrated that the surface of the co-encapsulated nano-liposomes was pegylated. Nano-liposomes could be coated with hydrophilic polymers like PEG, which gives them long-circulating properties (22, 23). The most significant finding of this study was that co-encapsulated nano-liposomes with good silibinin and glycyrrhizic acid content and suitable stability could be successfully developed.

*In vitro* cytotoxicity showed that the IC_50_ of coencapsulated nano-liposomes was lower than free silibinin (25% w/v) and glycyrrhizic acid (75% w/v). Toxicity tests indicated that cytotoxicity of the drug nano-carrier was approximately three times greater than the cytotoxicity of free drug (24). Silibinin was shown to seriously synergize the remedial efficacy of doxorubicin in advanced prostate cancer cells (25). An *in vitro* study showed that nano-liposome encapsulation of silibinin with glycyrrhizic acid increased the biological activity of free drugs, increased the stability of silibinin, and synergized the therapeutic effect of silibinin with glycyrrhizic acid on an HCC cell line.

## Conclusion

The results showed significant improvement in the co-entrapment of silibinin and glycyrrhizic acid to pegylated nano-liposome which had suitable EE, stability, controlled release rate of the drugs, good surface zeta potential and suitable mean diameter (less than 50 nm). The results have shown that the least IC_50_ belonged to co-encapsulated pegylated nanoliposomal silibinin and glycyrrhizic acid was less than that of free silibinin (25% w/v) and glycyrrhizic acid (75% w/v) on the HepG2 cancer cell line. Its usage is suggested for *in vivo* experiments.
